# Development of digital therapeutics in Hwa-byung treatment: exploring innovation potential in Korean medicine through practitioner survey

**DOI:** 10.3389/fmed.2024.1512337

**Published:** 2025-01-15

**Authors:** Chan-Young Kwon

**Affiliations:** Department of Oriental Neuropsychiatry, College of Korean Medicine, Dong-Eui University, Busan, Republic of Korea

**Keywords:** Hwa-Byung, Korean medicine, digital therapeutics, mind–body modalities, psychotherapy, survey, clinical practice

## Abstract

**Introduction:**

Hwa-byung (HB) is a culture-bound anger syndrome prevalent in Korea. While clinical practice guidelines emphasize mind–body modalities (MBMs) and psychotherapies for HB treatment, their implementation in Korean medicine (KM) remains unexplored. Digital therapeutics (DTx) offers potential solutions for treatment delivery barriers. This study investigated current HB treatment practices among KM doctors and their perceptions on DTx implementation.

**Methods:**

A nationwide web-based survey of licensed KMDs (*N* = 864) was conducted in South Korea (August–September 2024). The survey assessed HB treatment practices, utilization of MBMs and psychotherapies, and attitudes toward DTx. Data were analyzed using descriptive statistics.

**Results:**

Most KMDs utilized conventional KM treatments (acupuncture 95.4%, herbal medicine 94.0%), while MBMs (26.2%) and psychotherapies (21.3%) were underutilized despite their recognized importance. Primary barriers included time constraints (65.8%) and insufficient knowledge (62.9%). Treatment typically required 15.6 ± 60.7 weeks with 21.6 ± 37.4 sessions. While DTx knowledge was limited (mean score 2.3 ± 0.9/5), most KMDs (70.6%) expressed interest in DTx development, with 65.4% willing to implement it in practice. The estimated appropriate cost for HB DTx was 24,441.5 ± 13,566.0 KRW per session.

**Conclusion:**

This study reveals a significant gap between guidelines and clinical practice in HB treatment, particularly in implementing MBMs and psychotherapies. The positive attitude toward DTx suggests its potential as a practical solution to bridge this gap by providing accessible, standardized delivery of evidence-based psychological interventions within existing clinical workflows. Future DTx development should focus on addressing identified barriers while maintaining alignment with KM principles.

## 1 Introduction

Hwa-byung (HB), known as a “fire disease” or “anger syndrome” is a culture-bound syndrome prevalent in South Korea (1). The disorder is characterized by both psychological symptoms (including persistent suppressed anger, feelings of unfairness, and difficulty in emotional regulation) and somatic manifestations (such as chest tightness, hot sensations, headaches, and epigastric mass sensation) (1). These symptoms typically develop in response to chronic interpersonal conflicts where cultural norms discourage direct expression of anger (2). According to epidemiological studies, the prevalence of HB in South Korea varies by age and sex, and ranges from 1.5 to 12% (3). The core pathology of HB is suppressed anger, which is closely related to unfairness, and it was recently reported that the prevalence among the younger generation in South Korea, known to be sensitive to unfairness, is about 36% (4). According to HB clinical practice guidelines (CPG), this mental disorder is caused by unresolved and accumulated feelings of unfairness and resentment about stressful events (5). A recent study also reported a genetic vulnerability to HB, which is related to somatization (6). The identification of HB originated from Korean medicine (KM), East Asian traditional medicine, and KM doctors (KMDs) who currently treat this mental disorder (5). In South Korea’s dual healthcare system, KMDs are licensed medical professionals who practice KM after completing a 6-year medical curriculum and passing the national licensing examination. KMDs are legally recognized healthcare providers who can diagnose, treat, and prescribe traditional medications independently, parallel to conventional medical doctors (7).

The HB CPG recommends acupuncture, herbal medicine, moxibustion, mind–body modalities (e.g., the emotional freedom technique), and psychotherapy (e.g., acceptance and commitment therapy) as treatment methods for HB (5). Importantly, the CPG emphasizes the importance of mind–body modalities (MBM) and psychotherapies in the comprehensive treatment and eventual cure of HB (5). This recommendation is based on the understanding that HB, as a complex psychosomatic condition, requires interventions addressing both psychological and somatic components (2). However, the extent to which these recommended practices has been implemented in clinical settings remains unclear.

In recent years, interest in East Asian traditional medicine has been growing with modern technological advancements in healthcare (8). Digital therapeutics (DTx), an evidence-based therapeutic intervention driven by high-quality software programs, has emerged as a promising healthcare field (9). DTx offers the potential to deliver personalized and scalable interventions for various health conditions, including mental-health disorders (9). In the context of HB, DTx could potentially provide a means to bridge the gap between guideline recommendations and clinical practice, particularly in the delivery of MBMs and psychotherapies. Our research team is currently developing a DTx that provides evidence-based MBM and psychotherapy for HB.

While previous studies have documented HB’s prevalence (3, 4), clinical features (1), and recommended treatments (5), the real-world implementation of evidence-based interventions remains unexplored. This investigation addresses this knowledge gap by examining current clinical practices and treatment delivery barriers. This study aimed to fill this knowledge gap by conducting a comprehensive survey of KMDs in South Korea to investigate their clinical practices of HB and perceptions of DTx. In particular, the study focused on MBM and psychotherapy, which are essential for the fundamental treatment of HB (5). Specifically, this study aimed to investigate current HB treatment practices among KMDs, focusing on the implementation of guideline-recommended MBMs and psychotherapies, while exploring the potential role of DTx in addressing treatment delivery barriers. By examining both current practices and attitudes toward innovative solutions, this study provides insights for improving evidence-based HB care.

## 2 Methods

### 2.1 Participants

This study focused on licensed KMDs in South Korea. Participants were recruited with cooperation from the Association of Korean Medicine (AKOM). Specifically, the AKOM sent a survey link to its member doctors via email and text messages. The inclusion criteria were: KMDs who voluntarily participated in the survey, agreed to the academic use of their personal information, and were currently treating patients with HB in clinical practice. The survey referenced HB as ‘Hwa-byung, a common mental disorder in Korean medicine clinical settings.’ As all participants were licensed KMDs with standardized medical training in HB’s diagnosis and treatment, a detailed operational definition was not necessary for this professional population.

### 2.2 Questionnaire construction

A structured questionnaire consisting of four sections was developed based on a literature review (1–3, 5). The questionnaire was pilot tested with four KMDs, leading to minor revisions in question wording and response options to improve clarity.

#### 2.2.1 Demographic and professional characteristics

This section gathered information about the respondents’ age, sex, years of clinical experience, practice location, type of practice setting, and specialized training in psychiatry or related fields.

#### 2.2.2 Current clinical practices in Hwa-byung treatment

This section assessed the respondents’ awareness and use of standardized diagnostic and assessment tools for HB. The questionnaire included questions about the average number of HB patients seen per week to gauge the respondents’ experience with HB treatment. We also investigated the types of KM treatments frequently used for HB such as herbal medicine, acupuncture, and moxibustion. Questions regarding the average treatment duration, number of sessions required, and out-of-pocket costs of HB treatment were included to enable understanding of the typical treatment course and economic aspects. In the Korean healthcare system, out-of-pocket payment includes both the patient’s copayment for insurance-covered treatments and full payment for non-covered treatments.

#### 2.2.3 Knowledge and attitudes digital therapeutics for Hwa-byung

This section assessed respondents’ general knowledge of DTx and their awareness of any developments in KM DTx. It explored attitudes toward the potential development and use of DTx for HB treatment, including perceived needs and intentions to use such technologies, if available. Questions about the perceived appropriate cost of HB digital therapeutics were also included to gauge economic expectations.

#### 2.2.4 Use of mind–body modalities and psychotherapies for Hwa-byung

This section explored the frequency of use of MBMs and psychotherapies for HB treatment. Those who used these approaches were asked about the reasons for their use, perceived effectiveness, and any challenges encountered. Implementation barriers, such as time constraints, lack of knowledge, or perceived ineffectiveness, were investigated for those who did not use MBMs or psychotherapies. When asking about MBMs, the survey provided specific examples based on the clinical practice guidelines for HB (5): relaxation techniques, meditation, and emotional freedom technique. This definition was used to ensure participants’ understanding aligned with current clinical practice recommendations.

### 2.3 Distribution and collection of questionnaires

The survey was conducted online using a web-based platform (https://www.moaform.com/). Invitations for participation were distributed via email or text message. The survey was open to respondents from August 19 to September 24, 2024.

### 2.4 Data entry

The response results collected on the web-based survey platform were downloaded in Excel (Microsoft, Redmond, WA, USA) format and analyzed. Using this platform, all potential personal information that was not previously defined was removed.

### 2.5 Statistical analysis

Descriptive statistics were used to summarize the demographic characteristics of the respondents and their responses to the survey items. Frequencies and percentages were calculated for categorical variables, while means, standard deviations (SDs), and medians with interquartile ranges were computed for continuous variables. Data normality was assessed using the Shapiro–Wilk test. For the Likert-scale items, both individual-response frequencies and mean scores were reported. Statistical analyses were performed using SPSS software (version 18.0; IBM Corp., Armonk, NY, USA).

### 2.6 Ethical consideration

The study protocol was approved by the Institutional Review Board of the Dongeui University Korean Medicine Hospital (IRB No. DH-2024-04; approved on 27 May 2024). This study was conducted in accordance with the guidelines of the Declaration of Helsinki. All survey responses were anonymized during data collection. The web platform automatically removed identifying information before data download. Participants provided informed consent electronically before accessing the survey.

## 3 Results

### 3.1 Participants characteristics

**Figure 1:**
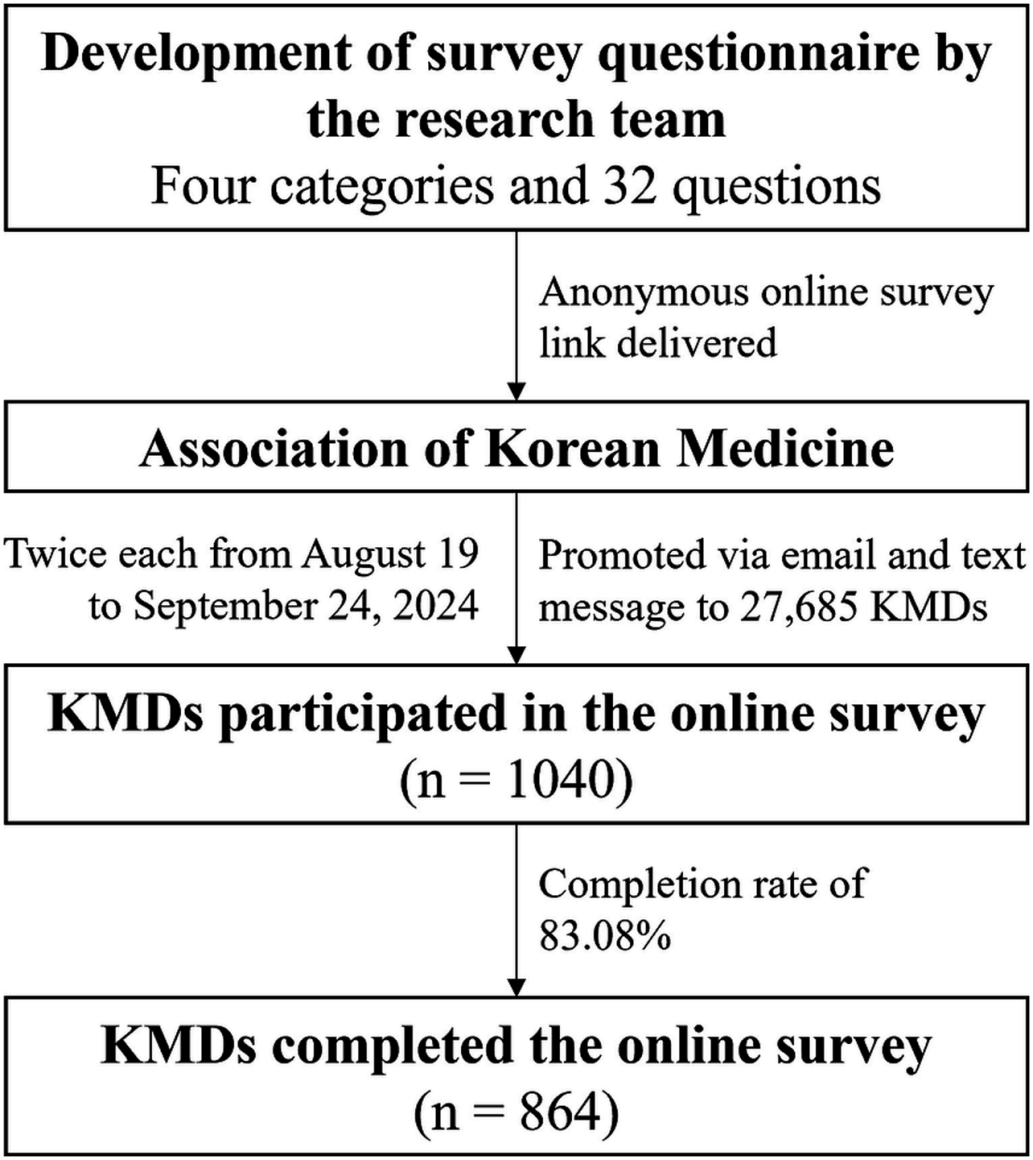
Flow chart of this survey. KMD, Korean medicine doctor.

In total, 864 KMDs completed the survey (Figure 1). The respondents’ sociodemographic characteristics are presented in Table 1. Most respondents were male (69.1%) and between the ages of 30 and 49 years (66.1%). Most had more than 10 years of clinical experience (69.6%) and worked in clinical settings (76.7%). Only 4.9% specialized in treating psychiatric patients in their clinical practice. The mean number of HB patients seen per week was 4.10 ± 6.62, with the majority (88.2%) seeing fewer than 10 patients with HB per week (Table 1).

**Table 1:** Sociodemographic characteristics of respondents.

Classification	Variables	*N* (%)
Sex	Male	597 (69.1%)
	Female	267 (30.9%)
Age (years)	< 30	49 (5.7%)
	≥ 30 to <40	280 (32.4%)
	≥ 40 to <49	291 (33.7%)
	≥ 50 to <59	203 (23.5%)
	≥ 60	41 (4.7%)
Clinical experience (years)	< 5	89 (10.3%)
	≥ 5 to <10	173 (20.0%)
	≥10 to <15	192 (22.2%)
	≥15 to <20	130 (15.0%)
	≥ 20 to <30	201 (23.3%)
	≥ 30	79 (9.1%)
Work area	Metropolitan area	477 (55.2%)
	Non-metropolitan area	387 (44.8%)
Work place	Clinic	663 (76.7%)
	Hospital	136 (15.7%)
	Nursing hospital	42 (4.9%)
	Public health center	23 (2.7%)
Advanced psychiatric practice	Yes	42 (4.9%)
	No	822 (95.1%)
Type of KMD license	Specialist	685 (79.3%)
	General practitioner	179 (20.7%)
Number of HB patients seen per week	< 10	762 (88.2%)
	≥ 10 to <20	78 (9.0%)
	≥20 to <30	12 (1.4%)
	≥ 30	12 (1.4%)
	Mean ± SD	4.10 ± 6.62; Median [IQR]: 2.0 [1.0–5.0]

HB, hwa-byung; IQR, interquartile range; KMD, Korean medicine doctor; SD, standard deviation.

### 3.2 Current status of Hwa-byung treatment for respondents

**Figure 2:**
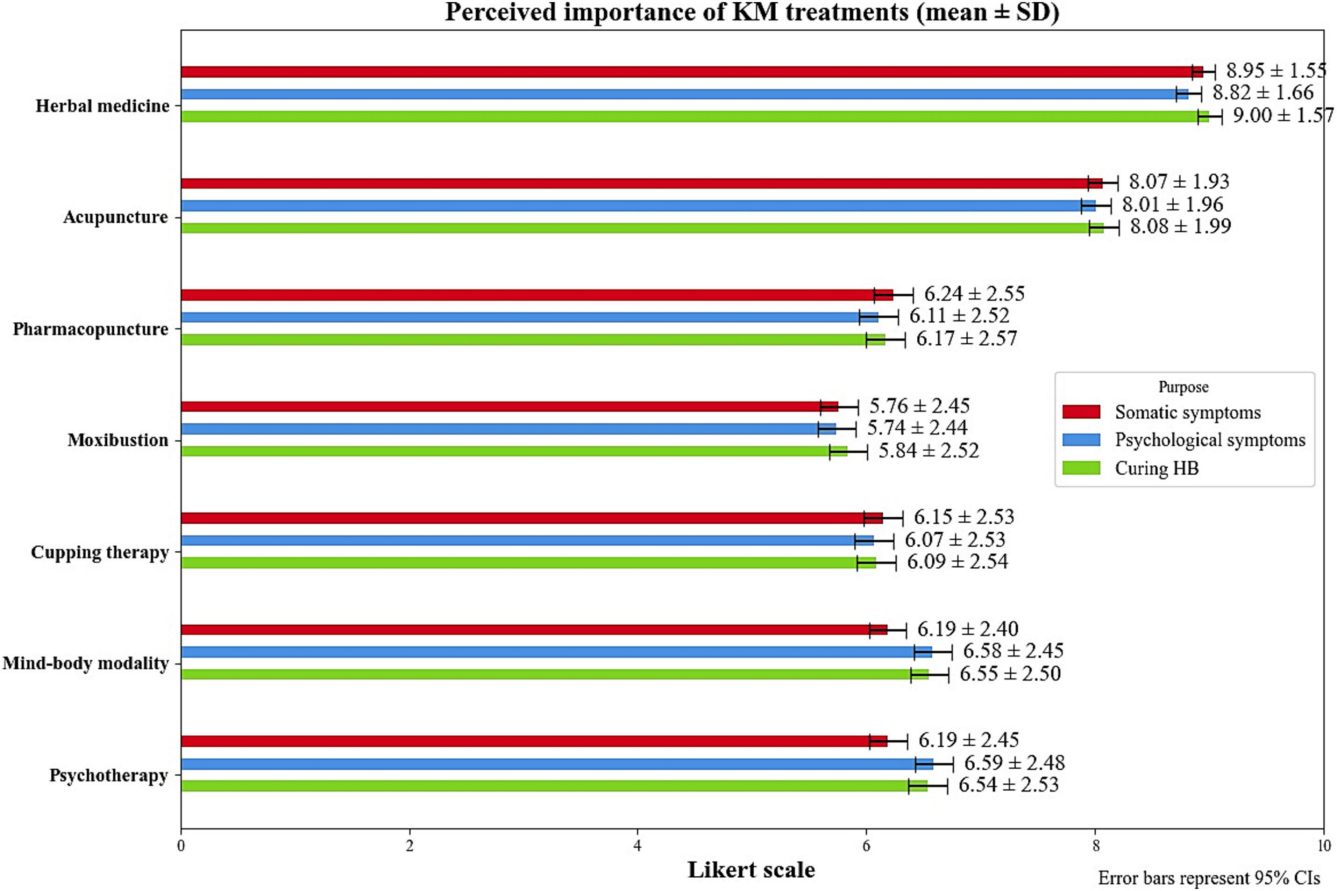
Perceived importance of KM treatments to treat HB. HB, Hwa-byung; KM, Korean medicine.

The majority of KMDs (67.2%) were unaware of standardized diagnostic tools for HB such as the Hwa-Byung Diagnostic Interview Schedule (HBDIS) (10), and among those who were aware, only 33.6% reported using it in their clinical practice. Similarly, 62.4% were unaware of standardized assessment tools for HB such as the HB scale (11), whereas only 35.1% were aware of these tools in clinical use. The mean duration of treatment required to cure HB was reported as 15.6 ± 60.7 weeks, with an average of 21.6 ± 37.4 treatments. The average out-of-pocket cost (including both insurance copayments and non-covered treatment costs) per session ranged mostly between 10,000 and 50,000 KRW (76.0% of the respondents) (Table 2). The most frequently used KM treatments for HB were acupuncture (95.4%) and herbal medicine (94.0%), followed by pharmacopuncture (35.5%), and cupping therapy (31.6%). Furthermore, MBMs (11.3%) and psychotherapy (7.8%) were used less frequently (Table 2). Notably, as KM treatments necessary for the complete cure of HB, MBMs and psychotherapy ranked third and fourth, respectively, after herbal medicine and acupuncture (Figure 2).

**Table 2:** Current status of HB treatment for respondents.

Classification	Variables	*N* (%)
Knowledge of the existence of standardized diagnostic tools for HB	Know	283 (32.8%)
	Do not know	581 (67.2%)
(If the respondent is aware of its existence) Use of standardized diagnostic tools for HB in clinical practice	Use	95 (33.6%)
	Do not use	188 (66.4%)
Knowledge of the existence of standardized assessment tools for HB	Know	325 (37.6%)
	Do not know	539 (62.4%)
(If the respondent is aware of its existence) Use of standardized assessment tools for HB in clinical practice	Use	114 (35.1%)
	Do not use	211 (64.9%)
KM treatments frequently used for the treatment of HB (3 multiple responses)	Herbal medicine	812 (94.0%)
	Acupuncture	824 (95.4%)
	Pharmacopuncture	307 (35.5%)
	Moxibustion	204 (23.6%)
	Cupping therapy	273 (31.6%)
	Mind–body modality	98 (11.3%)
	Psychotherapy	67 (7.8%)
	Other	7 (0.8%)
Average treatment cost (out-of-pocket) per treatment (KRW)	< 5,000	45 (5.2%)
	≥ 5,000 to <10,000	85 (9.8%)
	≥ 10,000 to <20,000	328 (38.0%)
	≥ 20,000 to <50,000	328 (38.0%)
	≥ 50,000 to <100,000	49 (5.7%)
	≥ 10,000	29 (3.4%)
Duration of treatment required to cure HB (weeks)	Mean ± SD	15.6 ± 60.7; Median [IQR]: 10.0 [6.3–12.0]
Number of treatments required to cure HB	Mean ± SD	21.6 ± 37.4; Median [IQR]: 18.0 [10.0–24.0]

HB, Hwa-byung; IQR, interquartile range; KM, Korean medicine; KRW, Korean Won; SD, standard deviation. 1 USD≒1,351 KRW (October 13, 2024).

### 3.3 Use of mind–body modality and psychotherapy for Hwa-byung

Only 26.2% of respondents reported using MBMs for HB in clinical practice. Among those who used MBMs, the main reasons were their effectiveness in improving HB psychological symptoms (66.4%) and overall psychological condition (56.2%). The primary reasons for not using MBMs were a lack of time (65.8%) and insufficient knowledge (62.9%). Similarly, only 21.3% of the respondents reported using psychotherapies for HB despite their recommended role in HB treatment. The main reasons for their use were similar to those of MBMs, with a focus on psychological symptom improvement (65.2%) and overall psychological condition enhancement (54.9%). The reasons for not using psychotherapy mirrored those for MBMs, with lack of time (63.8%) and insufficient knowledge (66.2%) being the primary barriers (Table 3).

**Table 3:** Use of MBM and psychotherapy for HB.

Classification	Variables	*N* (%)
Use of MBMs for HB in clinical practice	Use	226 (26.2%)
	Do not use	638 (73.8%)
(if the respondent use MBMs) Resons of the use (2 multiple responses)	Because it is effective in improving HB somatic symptoms	89 (39.4%)
	Because it is effective in improving HB psychological symptoms	150 (66.4%)
	Because it is effective in improving the patient’s overall physical condition (e.g., appetite, digestion, and sleep)	54 (23.9%)
	Because it is effective in improving the patient’s overall psychological condition (e.g., stress coping ability)	127 (56.2%)
	Because it is effective in fundamentally curing the disease	32 (14.2%)
	Other	0 (0%)
(If the respondent do not use MBMs) Resons of the use (2 multiple responses)	Because it is not effective in treating HB	40 (6.3%)
	Because there is not enough time to utilize it	420 (65.8%)
	Because there is not enough knowledge to utilize it	401 (62.9%)
	Because there is not enough materials to utilize it	270 (42.3%)
	Because patient compliance with it is poor	144 (22.6%)
	Other	11 (1.7%)
Use of psychotherapies for HB in clinical practice	Use	184 (21.3%)
	Do not use	680 (78.7%)
(If the respondent use psychotherapies) Resons of the use (2 multiple responses)	Because it is effective in improving HB somatic symptoms	59 (32.1%)
	Because it is effective in improving HB psychological symptoms	120 (65.2%)
	Because it is effective in improving the patient’s overall physical condition (e.g., appetite, digestion, and sleep)	55 (29.9%)
	Because it is effective in improving the patient’s overall psychological condition (e.g., stress coping ability)	101 (54.9%)
	Because it is effective in fundamentally curing the disease	33 (17.9%)
	Other	0 (0%)
(If the respondent do not use MBMs) Resons of the use (2 multiple responses)	Because it is not effective in treating HB	31 (4.6%)
	Because there is not enough time to utilize it	434 (63.8%)
	Because there is not enough knowledge to utilize it	450 (66.2%)
	Because there is not enough materials to utilize it	303 (44.6%)
	Because patient compliance with it is poor	131 (19.3%)
	Other	11 (1.6%)

HB, Hwa-byung; MBM, mind–body modality.

### 3.4 Knowledge and attitudes toward digital therapeutics for Hwa-byung

The general level of knowledge about DTx among KMDs was relatively low, with a mean score of 2.3 ± 0.9 on a 5-point scale. Knowledge about the development of KM DTx was similarly low (mean 2.2 ± 1.0). Despite the low level of knowledge, we observed a strong perceived need for the development of KM DTx for HB (mean 3.8 ± 0.8), with 70.6% of respondents indicating that such development was needed, or very needed. Additionally, 65.4% of respondents expressed an intention to use KM DTx for HB if they were developed (mean 3.7 ± 0.8) (Table 4). After removing outliers, the mean appropriate cost was estimated at 24,441.5 ± 13,566.0 KRW, ranging from 6,666 to 58,000 KRW (Table 5).

**Table 4:** Knowledge and attitudes toward DTx for treating HB.

Classification	Variables	*N* (%)
General level of knowledge about DTx	Do not know et al. = 1	168 (19.4%)
	Do not know = 2	338 (39.1%)
	Average = 3	279 (32.3%)
	Know = 4	62 (7.2%)
	Know well = 5	17 (2.0%)
	Mean ± SD	2.3 ± 0.9; Median [IQR]: 4.0 [3.0–4.0]
Knowledge level on the development of KM DTx	Do not know et al. = 1	203 (23.5%)
	Do not know = 2	371 (42.9%)
	Average = 3	201 (23.3%)
	Know = 4	74 (8.6%)
	Know well = 5	15 (1.7%)
	Mean ± SD	2.2 ± 1.0; Median [IQR]: 4.0 [3.0–4.0]
Need for the development of KM DTx for HB	Not needed at all = 1	13 (1.5%)
	Not needed = 2	25 (2.9%)
	Average = 3	216 (25.0%)
	Needed = 4	438 (50.7%)
	Very needed = 5	172 (19.9%)
	Mean ± SD	3.8 ± 0.8; Median [IQR]: 2.0 [2.0–3.0]
Intention to use KM DTx for HB	Never use = 1	13 (1.5%)
	Will not use = 2	27 (3.1%)
	Average = 3	259 (30.0%)
	Will use = 4	451 (52.2%)
	Will definitely use = 5	114 (13.2%)
	Mean ± SD	3.7 ± 0.8; Median [IQR]: 2.0 [2.0–3.0]

DTx, digital therapeutics; HB, Hwa-byung; IQR, interquartile range; KM, Korean medicine; SD, standard deviation.

**Table 5:** Appropriate cost of HB DTx.

Appropriate cost		*N*	Mean ± SD (KRW)	Range (KRW)
Raw data		864 (100%)	164,399.2 ± 689,804.1; Median [IQR]: 20,000.0 [10,000.0-50,000.0]	1 to 10,000,000
Outlier removal using *Z*-score of logged values	-3 < *Z*-score < 3	835 (96.6%)	170,108.7 ± 701,001.4; Median [IQR]: 20,000.0 [10,000.0-50,000.0]	20 to 10,000,000
	−2 < *Z*-score < 2	808 (93.5%)	99,059.1 ± 272,815.8; Median [IQR]: 20,000.0 [12,000.0-50,000.0]	200 to 2,000,000
	−1 < *Z*-score < 1	733 (84.8%)	31,628.0 ± 32,911.7; Median [IQR]: 20,000.0 [10,000.0-37,000.0]	2,000 to 2,000,000
	−0.5 < *Z*-score < 0.5	597 (69.1%)	24,441.5 ± 13,566.0; Median [IQR]: 20,000.0 [15,000.0-30,000.0]	6,666 to 58,000

DTx, digital therapeutics; HB, Hwabyung; IQR, interquartile range; KRW, Korean Won; SD, standard deviation.

## 4 Discussion

This comprehensive survey provides valuable insights into the current clinical practices of KMDs in HB treatment and their perceptions of DTx for HB management. The current study has several key findings. First, a significant proportion of KMDs were unaware of or did not use standardized diagnostic and assessment tools for HB. This suggests the need for better dissemination of these tools and training in their use, to enhance the standardization of HB diagnosis and treatment. Second, acupuncture and herbal medicine were the most commonly used treatments for HB, reflecting the strong roots of traditional KM practices. However, the limited use of MBMs and psychotherapies, despite their recommended role in HB CPG (5), is a significant finding. The low use of MBMs (26.2%) and psychotherapies (21.3%) for HB treatment, despite their emphasis in clinical guidelines, highlights a significant gap between evidence-based recommendations and real-world practice, and is a critical issue that must be addressed. This discrepancy may be attributed to the reported barriers of time constraints and a lack of knowledge, suggesting the need for targeted interventions to bridge this gap. Third, despite the limited knowledge of DTx, KMDs have shown a strong interest in their development and potential use for HB treatment. This positive attitude suggests a readiness to integrate innovative approaches into traditional practice, potentially as a means of bridging the gap between guidelines and practice in the clinical treatment of HB.

The findings of this study offer several important clinical implications for the treatment of HB in KM, particularly in the context of the existing literature (1, 3, 5). The low awareness and utilization of standardized diagnostic and assessment tools for HB (32.8 and 37.6%, respectively) highlights a critical gap in clinical practice (5). This result aligns with previous research highlighting the challenges in standardizing HB diagnosis and symptom assessment owing to its complex psychosomatic nature (1). Implementing these tools, including the HBDIS (10) and the HB scale (11) could enhance diagnostic accuracy and treatment efficacy, as evidence-based HB treatment studies have previously been conducted based on these standardized tools (5, 12).

The limited use of MBMs (26.2%) and psychotherapies (21.3%) for HB treatment, despite their emphasis on CPG (5), reveals a significant evidence-practice gap. Given that many MBMs, including tai chi, qigong, yoga, meditation, and relaxation, originate from East Asian traditional medicine systems (13), the low utilization of MBMs and psychotherapy by KMD for HB found in this survey is surprising. In the present survey, time constraints and knowledge gaps were identified as barriers to the use of MBMs and psychotherapy for the treatment of HB. Notably, among other reasons for not using MBMs and psychotherapy, five respondents responded that it was impossible to bill for these treatments. Since most MBM and psychotherapy are not billable medical procedures under the KMD in the current Korean medical system, this could result in the omission of these important treatments for HB. Addressing these barriers is essential, as comprehensive psychosomatic care has been shown to improve outcomes in conditions similar to HB (14, 15).

The strong interest in DTx among KMDs (70.6% perceived a need for its development) suggests a promising avenue for addressing identified practice gaps. This aligns with a growing body of evidence supporting the efficacy of digital interventions in mental health care (16). DTx could potentially serve as a bridge between KM treatments and evidence-based psychological approaches, helping to overcome the reported barriers of time and knowledge. DTx can be prescribed and billed by KM medical institutions without consuming excessive time (17). This characteristic potentially enables DTx to overcome the barriers that KMDs face when utilizing MBMs or psychotherapy for HB treatment. Consequently, the development of DTx, which provides evidence-based MBMs and psychotherapy for HB, may revolutionize the treatment of this culture-bound syndrome in South Korea.

Collectively, these findings suggest the need for a multifaceted approach to improving HB care in KM practice. This could include enhancing educational programs, developing time-efficient protocols for implementing psychosomatic interventions, and strategically integrating DTx to support evidence-based comprehensive HB treatment. Future research should focus on developing and validating such integrated approaches, potentially leading to more effective and standardized HB management protocols for KM.

This study has several limitations. First, the survey relied on self-reported information, which may have been subject to recall or social desirability biases. In addition, as a survey-based study, it provides broad insights, but may lack the depth of qualitative research methods in exploring KMD’s perspectives and experiences. Second, as a cross-sectional study, it cannot capture changes in practice patterns over time. Longitudinal research would be valuable to track the evolution of HB treatment approaches. Third, while efforts were made to obtain a diverse sample of KMDs, whether the sample is representative of the entire KMD population in Korea is not assured. Possibly, KMDs who were more interested in HB or DTx might have been more likely to participate in the survey, potentially influencing the results. While our sample size provides meaningful insights, qualitative methods could offer deeper understanding of KMDs’ perspectives and decision-making processes. Fourth, our survey provided only a basic reference to HB without a detailed operational definition, assuming a shared understanding among KMDs based on their standardized medical training. While this approach was appropriate for our professional study population, it could potentially lead to some variation in how participants conceptualized HB when responding to the survey. This limitation might be particularly relevant given the complex nature of culture-bound syndromes and their evolving understanding in clinical practice. Future studies might benefit from providing a standardized operational definition, even when surveying healthcare professionals, to ensure complete uniformity in the understanding of HB across participants. Fifth, as a quantitative study, our research has inherent limitations in deeply understanding the underlying reasons for identified barriers to implementing MBMs and psychotherapy. While our findings reveal key barriers, qualitative research would be valuable to explore the specific challenges KMDs face in their clinical settings. Additionally, although our sample includes diverse practice settings, future studies focusing on specific patient demographics and treatment environments could provide more detailed implementation insights.

## 5 Conclusion

This survey provided a comprehensive overview of the current clinical practices of KMDs in HB treatment and their perceptions of DTx. The findings revealed a strong reliance on KM treatments, with limited use of standardized tools, MBMs, and psychotherapies, highlighting a significant gap between HB CPG and real-world practice. Despite a low familiarity with DTx, we found a positive attitude toward its development and potential use in HB management. This openness to innovation presents a unique opportunity to address the identified practice gaps, particularly in the implementation of MBMs and psychotherapy for HB treatment. These results highlight several opportunities for improving HB care, including promoting standardized diagnostic and assessment tools, integrating psychological approaches as recommended by the guidelines, and developing targeted DTx. The identified barriers to the use of MBMs and psychotherapies suggest that DTx could play a valuable role in addressing these gaps, potentially serving as a bridge between evidence-based recommendations and clinical practice. Key steps to address these gaps include educational programs for KMDs on MBMs and psychotherapy implementation, and development of DTx solutions incorporating evidence-based approaches such as mindfulness and acceptance and commitment therapy. Future research should focus on evaluating specific DTx interventions that efficiently integrate these evidence-based treatments into clinical workflows. These digital solutions should emphasize the integration of MBMs and psychotherapies, as recommended by clinical guidelines.

## Data Availability

The raw data supporting the conclusions of this article will be made available by the authors, without undue reservation.
